# Quasi-static and dynamic mechanical properties of a linoleic acid-modified, low-modulus bone cement for spinal applications

**DOI:** 10.12688/openreseurope.16683.2

**Published:** 2024-05-21

**Authors:** Salim Ghandour, Iain Christie, Caroline Öhman Mägi, Cecilia Persson

**Affiliations:** 1 Division of Biomedical Engineering, Department of Materials Science and Engineering, Uppsala University, Uppsala, Uppsala County, 75121, Sweden; 2 Division of Applied Materials Science, Department of Materials Science and Engineering, Uppsala University, Uppsala, Uppsala County, 75121, Sweden

**Keywords:** PMMA, bone cement, vertebroplasty, discoplasty, fatigue, tensile properties

## Abstract

**Background:**

Polymethylmethacrylate (PMMA) bone cement is extensively used in spinal procedures such as vertebroplasty and kyphoplasty, while its use in percutaneous cement discoplasty (PCD) is not yet widely spread. A main issue for both application sites, vertebra and disc, is the mismatch in stiffness between cement and bone, potentially resulting in adjacent vertebral fractures and adjacent segment disease. Tailoring the cement modulus using additives is hence an interesting strategy. However, there is a lack of data on the tensile and tension-compression fatigue properties of these cements, relevant to the newly researched indication of PCD.

**Method:**

A commercial PMMA cement (VS) was modified with 12%vol of linoleic acid (VSLA) and tested for quasi-static tensile properties. Additionally, tension-compression fatigue testing with amplitudes ranging from +/-5MPa to +/-7MPa and +/-9MPa was performed, and a Weibull three-parameter curve fit was used to calculate the fatigue parameters.

**Results:**

Quasi-static testing revealed a significant reduction in VSLA’s Young’s Modulus (E=581.1±126.4MPa) compared to the original cement (E=1478.1±202.9MPa). Similarly, the ultimate tensile stress decreased from 36.6±1.5MPa to 11.6±0.8MPa. Thus, VSLA offers improved compatibility with trabecular bone properties. Fatigue testing of VSLA revealed that as the stress amplitude increased the Weibull mean number decreased from 3591 to 272 and 91 cycles, respectively. In contrast, the base VS cement reached run-out at the highest stress amplitude. However, the lowest stress amplitude used exceeds the pressures recorded in the disc *in vivo*, and VSLA displayed a similar fatigue life range to that of the annulus fibrosis tissue.

**Conclusions:**

While the relevance of fully reversed tension-compression fatigue testing can be debated for predicting cement performance in certain spinal applications, the results of this study can serve as a benchmark for comparison of low-modulus cements for the spine. Further investigations are necessary to assess the clinical feasibility and effectiveness of these cements.

## Introduction

Acrylic polymethylmethacrylate (PMMA) bone cement is one of the most commonly used biomaterials in orthopaedics ^1, 2^. It is used extensively in the spine for the treatment of vertebral compression fractures (VCFs) through vertebroplasty ^3^ and kyphoplasty procedures ^4, 5^, where the cement is injected minimally invasively into the fractured vertebrae, providing stability and pain relief once it is set *in situ*. More recently, PMMA cement has been tried for certain patients suffering from advanced disc degeneration disease (DDD), through a procedure named percutaneous cement discoplasty (PCD) ^6^.

However, patients that suffer from VCFs and DDD in the elderly population often suffer from osteoporosis ^7, 8^, and PMMA typically has an elastic modulus of around 1700 to 3700 MPa ^9, 10^ while the trabecular bone found inside a vertebra varies between 10–900 MPa ^11, 12^, with the lower values stemming from osteoporotic patients. This difference elevates the risk of adjacent vertebral fractures (AVFs), in particular for osteoporotic patients. Clinical and radiological studies have revealed that the incidence of adjacent vertebral fractures following vertebroplasty ranges from 54% to 67% ^13, 14^ and 30% to 90% after kyphoplasty ^15^. As for PCD, risks of AVFs have been expressed ^16^, but current studies suggest a low number of patients suffering from AVFs ^17^. However, osteoporotic patients are not currently considered for such surgeries ^18, 19^.

Tailoring the properties of PMMA to match those of the surrounding bone would reduce the risk of AVFs in vertebroplasty and kyphoplasty, while also potentially enabling its use in PCD procedures for osteoporotic patients. Notably, a finite element simulation of loading after discoplasty revealed reduced stresses on the endplates when utilizing a low-modulus cement ^20^. This suggests the potential to improve stress distribution and reduce the risk of adjacent fractures as well as adjacent segment disease (ASD) in PCD patients.

The development of low-modulus PMMA has been extensively investigated in vertebroplasty to accommodate for the elastic mismatch between the PMMA and bone ^21, 22^. This type of PMMA contains additives that may *e.g.* act as plasticizers ^23^ or a gel that creates a porous cement ^24^ with the aim of reducing the elastic modulus. For example, fatty acids ^25^, cellulose derivatives ^26, 27^, N-methyl-pyrrolidone ^28^, and mixtures of blood and saline solutions have previously been investigated ^29, 30^. One such additive is linoleic acid (LA). Linoleic acid is a type of fatty acid that has been shown to reduce the mechanical properties of PMMA even when added in small amounts, thereby maintaining similar handling properties to standard cements. The addition of LA affects the polymerisation reaction by creating competing reactions, leading to shorter polymer chains but also a lower polymerization temperature, beneficial for the surrounding tissues ^31, 32^. Furthermore, LA acts as a plasticizer, reducing the friction between adjacent polymer chains ^31^. In previous work by the authors, the addition of LA to PMMA cements has been thoroughly characterized in terms of bench, *in vitro* and *in vivo* properties. Robo *et al.* found that the addition of 12%vol LA to commercial V-steady™ PMMA (G21, Italy) reduced the elastic modulus from 2140.4 ± 128.8 MPa to 494.7 ± 51.8 MPa ^31, 33^ and that it was the optimal volume to add to, in the long-term, obtain mechanical properties similar to vertebral trabecular bone ^33^. In addition, compression-compression fatigue testing has showed that this type of cement has a fatigue limit of 2.5–5 MPa after 2 million cycles (where it can be noted that the fatigue limit was defined as a decrease in sample height of 15%, the clinical definition of a vertebral compression fracture, but the samples did not fracture) ^31, 33^. These results are comparable to pressures found in the disc of 0.27–2.3 MPa during daily activities ^34^. Additionally, an *in vivo* study in a sheep model observed similar inflammatory response between the commercial F20® cement (Teknimed S.A.S, France) and the LA-modified version of this cement. This suggests that the addition of LA does not negatively affect the bone remodelling process around the implant ^35^. Finally, LA has been shown to possess antibacterial properties ^36, 37^. The above listed advantages with LA, and its advancement along the value chain, made it the component of choice for the present study. Currently, no other low-modulus PMMA cements are in clinical use, to the best of the authors’ knowledge.

While compression-compression fatigue tests show promise for use in the envisaged end applications ^33^, there is no data on the cement’s tensile behaviour, nor on its behaviour under tension-compression fatigue. This type of data is considered important for benchmarking purposes, as it is part of the test battery in the ASTM F2118 standard for assessment of PMMA bone cement ^38^. It may also be of higher importance for the novel PCD application as the disc is typically exposed to bending and hence parts of it to tension ^39^. Therefore, the aim of this study was to evaluate the tensile properties as well as the tension-compression fatigue properties of the novel low-modulus bone cement, according to ASTM F2118.

## Methods

### Materials

V-Steady bone cement (VS) (G21 srl., Italy) was used as a control as well as the base cement for making the low-modulus bone cement in this study. The solid phase was composed of 14.1g of PMMA, 0.2g of benzoyl peroxide (BPO), and 11.7g of zirconium dioxide, while the liquid phase contained 9.6g of methyl methacrylate, 0.13g of N.N dimethyl-p-toluidine, 50 ppm of hydroquinone. Further, 12 vol% of linoleic acid (LA, 99%, Sigma-Aldrich, St. Louis, MO, USA) was added into to liquid phase ahead of cement mixing for the low-modulus cement (VSLA).

### Preparation of the cement samples

The cements were prepared according to manufacturer’s instructions. The liquid and solid phase were mixed in a glass mortar using a spatula for 30–60 seconds at room temperature. The cement was then poured into a syringe from which silicon moulds of the desired dog-bone shape were filled. The dimensions of the moulds were designed according to ASTM F2118. The gauge section of the sample was 10mm long and 5mm in diameter while the thicker, grip section was 8.5mm in diameter. A total of 30 samples were used for quasi-static tensile testing, i.e. n=15 per group VS and VS-LA. At least 15 samples per fatigue sample group were also used, as required by the ASTM F2118 standard. After preparation, the samples were then allowed to set in these moulds for 24 hours in phosphate buffer saline (PBS) solution (PBS, Sigma-Aldrich, St. Louis, Mo, USA) at 37°C, before being removed from the moulds and being further conditioned for another 13 days in PBS at 37°C. Previous studies have reported that the mechanical properties of the cement plateau at around 2 weeks ^20^.

### Quasi-static tensile testing

An MTS 858 Mini Bionix (MTS Systems Corporation, United States) was used to estimate the ultimate tensile strength (UTS) and Young’s modulus of the bone cement under tension loading. Hydraulic clamps were used to fix the samples onto the machine. The cross-head speed used was 5mm/min and the force and displacement of the sample were recorded. In order to accurately estimate the Young’s modulus of the sample, the test was recorded by video and a custom MATLAB (RRID:SCR_001622) script was developed to analyse the movement of two markers on the gauge length of the sample, acting as an optical extensometer. Alternatively, this script can be written in Python (RRID:SCR_008394) with relevant packages such as NumPy (RRID:SCR_008633).

Each sample was marked with two black lines 10mm apart to isolate the sample gauge. The tests were then recorded at 60fps using a 1080p camera, each image was extracted from the video and analysed within MATLAB in greyscale. Each frame was labelled numerically in ascending order. An Otsu filter with a threshold level of 0.3 was applied in MATLAB to the raw image in order to create a binary black and white image for each frame. Each frame was then rotated 90 degrees for processing purposes. Once every frame had been extracted, filtered and rotated, a region of interest (ROI) was identified by the code. The initial ROI was identified by the user manually inserting the approximate positions of the middle, top and bottom of the sample. From this each column of pixels is analysed until a solid black line of pixels, the length of which is defined by user input, is identified on either side of the image. The distance between these two lines defines the initial length of the sample as well as the initial ROI. To calculate the strain, the initial length of this ROIs compared to the length of the region of interest in each proceeding frame in the video. From this, the change in length of the ROI and hence the strain was calculated. The Young’s modulus of all the samples was then calculated between 0.2% and 0.4% strain to ensure consistency and remain within the linear region of the curve.

### Tension-compression fatigue testing

The MTS 858 Mini Bionix was also used to test the properties of the bone cement under fully reversed tension-compression fatigue (R=-1) at the three stress levels defined in the ASTM F2118 standard, *i.e.* +/-5, 7 and 9 MPa ^38^. For these tests, the setup was surrounded by a biobath which was filled with circulating PBS solution at 37°C, since previous studies have observed an effect of the environment on the fatigue performance ^40^. The samples were cycled to failure at their respective stress levels and the cycles to failure were recorded. A Weibull distribution at each stress level was then calculated using a custom MATLAB script. The three-parameter Weibull distribution is commonly used to effectively analyse fatigue data ^9, 41, 42^. The three-parameter Weibull distribution is given by ^43^:


$$\;R(N_{f})=-exp\left[-{\left(\frac{N_{f}-N_{0}}{N_{a}-N_{0}}\right)}^{b}\right]\;(1)$$

Where *R*( *N _f_* ) is the survival probability of the sample, *N _f_* is the loading cycle, *N*_0_ is the minimum or guaranteed fatigue life, *N _a_* is the characteristic fatigue life, *b* is the Weibull slope.

where *R*( *N _f_* ) is determined from the expression:


$$\;R(N_{f})=\frac{M-0.3}{G+0.4}\;(2)$$

Where M is the assigned rank of sample after the data is arranged in ascending order of magnitude, and G is the total number of specimens. After calculating *R(N _f_)* for all samples, initial parameters for the Levenberg-Marquardt algorithm are calculated. *N*_0_ is determined by finding the asymptote of the best fit line for the plot of $\ln\;(\ln(\frac{1}{R(y)}))$ against In ( *N*_*x*_). The Weibull shape factor ( *b*) is derived from the gradient of the best fit line for the graph ln ( *N*_*x*_ – *N*_*o*_) versus the linearised Weibull probability.

Once all variables are calculated they can be substituted into the linearized Weibull equation ( Equation 3) to find the Weibull characteristic fatigue life ( *N*_*a*_).


$$b[\ln(N_{x}-N_{o})]-b[\ln(N_{a}-N_{o})]=\ln\left(ln\left(\frac{1}{P(x)}\right)\right)\;(3)$$

Then, these parameters are used as a starting point for the Levenberg–Marquardt non-linear regression method (Curve Fitting Toolbox™ in MATLAB version R2022a; The MathWorks® Inc., Natick, MA, USA), which then calculated optimised *N*_0_, *N _a_*, and *b* estimates to reduce the errors of the fit.

These optimised Weibull estimates were then used to compute the Weibull mean fatigue life ( *N*_*WM*_).


$$N_{WM}=N_{0}+(N_{a}-N_{0})\text{Γ}(1+1/b)\;(4)$$

Where *N _WM_* is the Weibull mean number of fatigue cycles, and Γ is the gamma function. The Weibull distributions and parameters were calculated for the low modulus bone cement at stress amplitudes of +/-5 MPa, +/-7 MPa and +/-9 MPa.

### Statistical analysis

The statistical analysis was performed using Prism GraphPad 10 (RRID:SCR_002798). Normality of the data was checked using Q-Q plots. An unpaired t-test was conducted to assess any differences between elastic modulus and ultimate tensile strength of VS and VSLA. A significance level of α=0.05 was used.

## Results

### Properties under quasi-static tensile load

A total of 30 samples were tested for VS (n=15) and VSLA (n=15) cements. The Young’s modulus and UTS for the control VS cement were found to be 1478.1 ± 202.9 MPa and 36.6 ± 1.5 MPa, respectively, both of which were found to substantially decrease for the VSLA cement, as expected, giving a Young’s modulus of 581.1 ± 126.4 MPa and an UTS of 11.6 ± 0.8 MPa ^44^ ( Figure X).

**Figure X.**
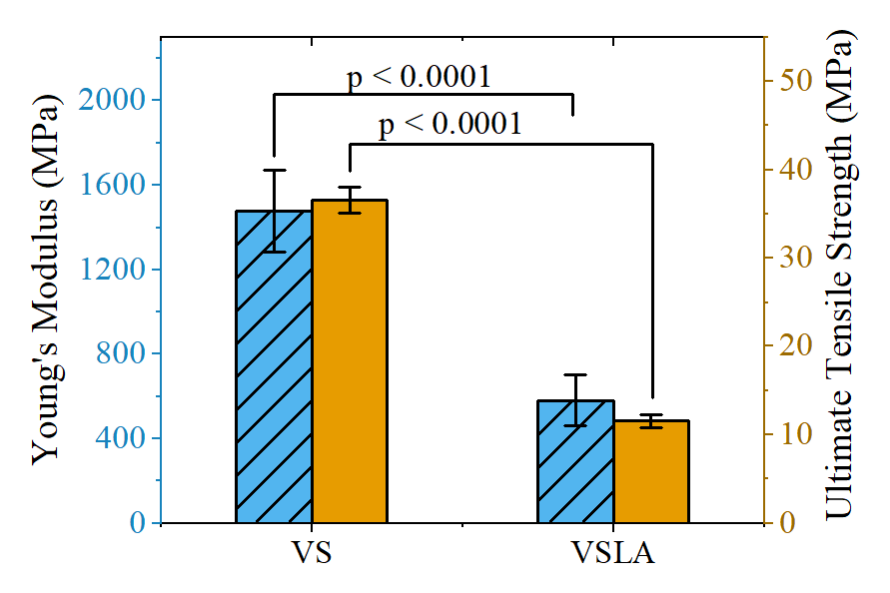
Quasi-static tensile properties of VS and VSLA cement.

### Properties under tension-compression fatigue

A total of 47 VSLA samples were prepared for dynamic testing. At least 15 samples were used for each stress amplitude ( Table 1).

**Figure 1.**
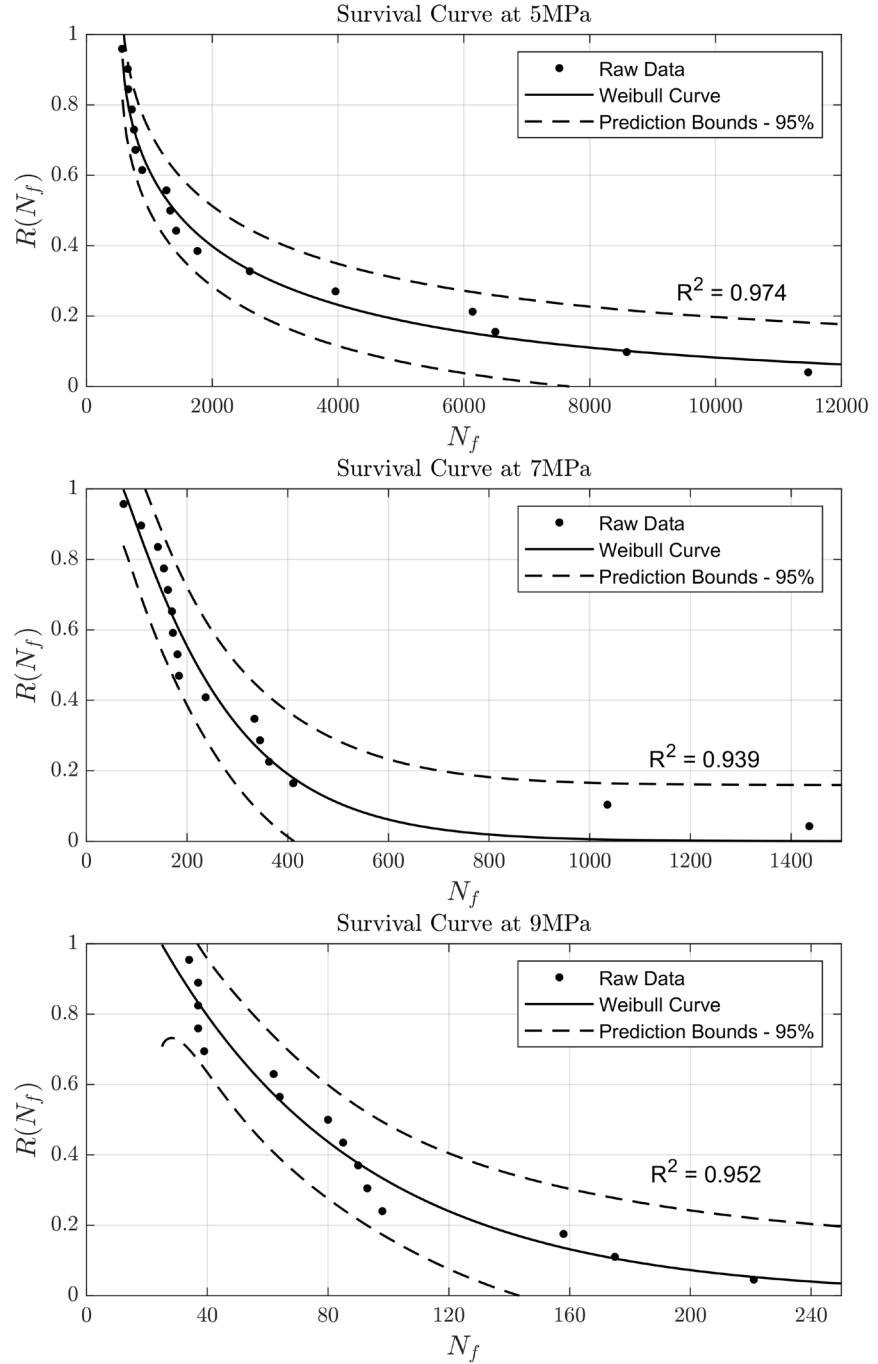
Survival Weibull curves for VSLA at 5 MPa, 7 MPa, and 9 MPa. Dashed lines indicate 95% confidence interval of the fitting.

The data points and Weibull survival curves for VSLA are presented in Figure 1, showing good fits (R ^2^>0.93) to the Weibull model.

The Weibull parameters associated with the low-modulus cement including: the Weibull characteristic fatigue life ( *N _a_*), the estimated minimum fatigue life ( *N _o_*), the Weibull mean number ( *N _wm_*), and the Weibull slope ( *b*) are summarised in Table 1. As expected, the minimum and mean fatigue life values decreased with an increase in test amplitude. Conversely, the Weibull slope increases with increasing stress. This indicates that the sample is more likely to survive more cycles at lower stress levels.

Additionally, three VS samples were tested at 9 MPa and reached a run-out of 5 million cycles (run-out value taken according to the standard ^38^).

**Table 1.** The Weibull parameters associated with the low-modulus cement.

	N _o_ (cycles)	N _a_ (cycles)	N _wn_ (cycles)	b
**5MPa (n=17)**	564	2246	3591	0.53
**7MPa (n=15)**	73	279	272	1.09
**9MPa (n=15)**	25	91	91	1.00

## Discussion

The objective of this study was to determine the mechanical properties of a low-modulus PMMA bone cement in tension and tension-compression fatigue. This was carried out using the ASTM F2118 standard ^38^. A commercial cement was used as a control to study the effectiveness of using LA to reduce the mechanical properties under these loading scenarios, and to benchmark its performance in relation to such a cement.

The mean tensile properties of the bone cement were significantly reduced with the addition of linoleic acid with the Young’s modulus decreasing by 61%, from 1478.13 ± 202.92 MPa to 581.10 ± 126.44 MPa, and the mean UTS decreasing by 68%, from 36.57 ± 1.54 MPa to 11.57 ± 0.77 MPa. As discussed previously, the mechanism behind this reduction of mechanical properties is that LA serves as a plasticizer, reducing friction between adjacent polymer chains, while also affecting the polymerization process by initiating competing reactions, resulting in shorter polymer chains and some increase in unreacted monomer ^33^. The UTS of the modified PMMA was still significantly higher than that of vertebral trabecular bone, which has a UTS between 1.33–3.53 MPa in the inferior-superior direction ^12^. The Young’s modulus of the cement was significantly reduced, in accordance with the additive’s aim of achieving a low-modulus cement. The Young’s modulus of VSLA was within the range of moduli recorded for human trabecular bone (1–976 MPa), implicating that the novel cement is better suited for the target treatments, in terms of a reduced risk for adjacent VCFs when compared to commercial PMMA (1700–3500 MPa) ^9, 10^. A study by Weightman *et al.* developed a low-modulus PMMA formulation, suggesting that such cements can result in more even distribution of the stresses in the cement and thereby reducing the risks of fracture, although for the case of fixation of hip replacements ^45^. Their developed cement was compared to previously used commercial cements CMW and Sulphix using tensile testing. The commercial cements had Young’s modulus and UTS ranging between 1330–1820 MPa and 36–43 MPa, respectively, while the low modulus cement had a mean Young’s modulus and UTS of 650 MPa and 19 MPa, respectively ^45^. Although the applications are different, the results and aim of this low modulus cement correlate well with the intended reduction of mechanical properties observed with VS and VSLA. The modulus of the intervertebral disc varies depending on the region of the disc measured, the manner in which the disc is measured, and the stage to which the disc is degenerated ^46^. Even at the highest end of the disc moduli, 140 MPa when the annulus fibrosis was measured in tension ^47^, the bone cement has a much higher modulus than the disc. However, one also needs to consider that under compression, a healthy nucleus exhibits non-linear and viscoelastic properties ^39^, and an exact match to the complete disc properties is difficult to reach in one and the same material. Despite this, the reduced cement modulus could still aid in reducing the incidence of adjacent segment disease – resulting in the cement potentially being better suited to PCD than currently available bone cement. Indeed, the UTS of the modified PMMA was in the higher range of the annulus UTS, which ranges between 6–11 MPa ^48^, and, as previously mentioned, an earlier finite element study indicated that a lower modulus cement could reduce the pressure in adjacent tissues ^20^. The latter however remains to be confirmed in *in vivo* studies and clinical trials.

The fatigue properties of the cement were examined using a Weibull distribution, which was obtained for each of the stress amplitudes defined by the standard, *i.e.* ±5 MPa, ±7 MPa and ±9 MPa. As expected, the distributions seen in Figure 1 show that as the stress amplitude increases, the average number of cycles to failure drops. The Weibull characteristic fatigue life ( *N _a_*), the estimated minimum fatigue life ( *N _o_*) and the Weibull mean number ( *N _wm_*) values seen in Table 1 can be compared to values in the literature, summarized in Table 2. Unfortunately, there is a lack of data for commercially available cements for vertebroplasty, and comparisons are only made to bone cements available for joint arthroplasty, which are to be tested at other stress levels according to the standard (10 MPa, 12.5 MPa, and 15 MPa). Table 2 shows that the Weibull characteristic fatigue life ( *N _a_*), the estimated minimum fatigue life ( *N _o_*) and the Weibull mean number ( *N _wm_*) values are all significantly lower for the low-modulus cement compared to the other commercially available cements.

**Table 2.** Characteristic Weibull parameters of different cements. R-O indicates run-out. N/A = data not available.

Cement	Test Type	Frequency (Hz)	Stress (MPa)	N _a_ (Cycles)	N _o_ (Cycles)	N _wm_ (Cycles)
**VSLA (n=17)**	Compression-Tension	2	+/- 5	2246	564	3591
**VSLA (n=15)**	Compression-Tension	2	+/- 7	279	73	272
**VSLA (n=15)**	Compression-Tension	2	+/- 9	91	25	91
**VS (n=3)**	Compression-Tension	2	+/- 9	R-O	R-O	R-O
**Simplex P** ^41^	Compression-Tension	1	+/- 15	20901	4915	24218
**Orthoset 1** ^41^	Compression-Tension	1	+/- 15	24780	8103	25280
**Orthoset 3** ^41^	Compression-Tension	1	+/- 15	108659	13360	118951
**Unspecified commercially** **available cement** ^42^	Compression-Tension	2	+/- 10	N/A	N/A	610679
**Simplex P** ^9^	Compression-Tension	2	+/- 10	221606	N/A	N/A

However, it is important to highlight that the minimum stress tested in this study was ±5 MPa, which is far larger than the stresses measured in the IVD while walking and running, 0.59 MPa and 0.65 MPa respectively ^49^. Considering that vertebrae are mainly under compression loading, the relevance of the loading case suggested by the ASTM F2118 could be debated, especially for vertebroplasty and kyphoplasty ^34, 50^. In contrast, discs may undergo tension and compression depending on the loading case. Tension is predominantly observed in the annulus of the disc due to swelling and bending. It has been reported that the bulk annulus fibrosis has a UTS between 0.9 to 8.6 MPa depending on its location ^51^. Notably, fatigue failure occurred in annulus samples in less than 10,000 cycles when the forces exceeded 45% of the UTS ^48^. Similarly, VSLA has an UTS of around 11.6 MPa and fails in less than 12,000 cycles ( Figure 1) at a stress level of approx. 43% of UTS (5 MPa) and above – suggesting a similarity in mechanical performance of VSLA to the disc tissue, although conclusions can only be limited to a similar order of magnitude. More comprehensive testing would need to be conducted to confirm the appropriate functionality of these cements for the use in PCD, although the higher UTS of the VSLA compared to the maximum UTS measured for the annulus fibrosis is promising.

This study has some limitations. The virtual extensometer is sensitive to the lighting conditions of the room. It is important to ensure ambient lighting and that camera exposure is kept constant throughout the testing duration. However, this would only have a potential effect on the Young’s modulus values reported, in terms of a somewhat larger spread in data. Regarding future work, the antibacterial properties of LA have been discussed previously but have not yet been studied within a cement. Exploring the synergistic effects of adding LA to PMMA cements is an area of interest particularly with the increasing concerns on antimicrobial resistance ^37^. Further, the mechanical effects and benefits of soft cements in PCD should be characterised using established *ex-vivo* models ^52, 53^ before further advancement to clinical studies.

## Conclusions

In conclusion, this study closed a knowledge gap by providing insights into the quasi-static tensile and tension-compression fatigue properties of a novel linoleic-acid containing, low-modulus bone cement for spinal treatments. The findings demonstrated that the modified cement exhibited a significant reduction in Young’s Modulus and ultimate tensile stress compared to the original cement, indicating improved compatibility with trabecular bone mechanical properties, which could be beneficial for reducing the number of adjacent vertebral fractures after treatments. Tension-compression fatigue testing showed a decrease in survival rates as stress amplitude increased from +/-5 to 7 to 9 MPa, and a much lower fatigue life in tension-compression than standard, commercially available cements for vertebroplasty and joint arthroplasty. However, the lowest stress amplitude used widely exceeds pressures recorded *in vivo* in the disc, and the cement exhibited a similar fatigue life range to that of the annulus fibrosis tissue found in the disc. While the results provide a benchmark for comparing low-modulus cements for spinal applications, further research is needed to evaluate the clinical feasibility and effectiveness of these bone cements.

## Data Availability

Zenodo: Raw mechanical testing data https://doi.org/10.5281/zenodo.8380702 ^44^ This project contains the following underlying data: FatigueData.zip (contains all the fatigue data for the VSLA cement) TensileData.zip (contains all the tensile testing data for the VS and VSLA cement) VS videos.zip (contains the videos used to calculate tensile properties for the VS cement) VSLA videos.zip (contains the videos used to calculate the tensile properties for the VSLA cement) CorrectedTensileData.xlsx (contains all the information on which samples were used to calculate the tensile properties) README.txt (contains a summary of each folder and abbreviations used)

## Software availability

Software available from: https://mathworks.com

Source code available from: https://github.com/salgh/OpticalExtensometerUU.git

Archived source code at time of publication: https://doi.org/10.5281/zenodo.10016901 ^54^

License: BSD-3-Clause
