# Supplementary material for: Quasi-static and dynamic mechanical properties of a linoleic acid-modified, low-modulus bone cement for spinal applications
Source: Open Res Eur. 2024 May 21;3:203. Originally published 2023 Nov 20. [Version 2] doi: 10.12688/openreseurope.16683.2 (PMC11344196; doi:10.12688/openreseurope.16683.2)
Supplement: Peer review history

# Reviewer report: Reviewer response for version 2

**DOI:** 10.21956/openreseurope.19102.r40756

**Published:** 2024-08-23

**Authors:** Jakub Szabelski

**Affiliations:** 1 Lublin University of Technology, Lublin, Poland

I have no additional comments.

Is the study design appropriate and does the work have academic merit?

Yes

Is the work clearly and accurately presented and does it cite the current literature?

Partly

If applicable, is the statistical analysis and its interpretation appropriate?

Partly

Are all the source data underlying the results available to ensure full reproducibility?

Yes

Are the conclusions drawn adequately supported by the results?

Yes

Are sufficient details of methods and analysis provided to allow replication by others?

Yes

Reviewer Expertise:

mechanical properties of bone cements, biomaterials,

I confirm that I have read this submission and believe that I have an appropriate level of expertise to confirm that it is of an acceptable scientific standard.

# Reviewer report: Reviewer response for version 2

**DOI:** 10.21956/openreseurope.19102.r40757

**Published:** 2024-06-18

**Authors:** Marcin Wekwejt

**Affiliations:** 1 Gdansk University of Technology, Gdansk, Poland

This manuscript, titled " *Quasi-static and dynamic mechanical properties of a linoleic acid-modified, low-modulus bone cement for spinal applications*" is an intriguing paper that focuses on the modification of PMMA bone cement with linoleic acid and its potential for adjusting cement stiffness, particularly for percutaneous discoplasty applications. The paper is well-conceived, presenting valuable findings in the field of quasi-static tension and tension-compression fatigue properties of composite cement and is entirely original. Furthermore, the article has undergone a major revision process. The authors have addressed all comments appropriately, corrected the main shortcomings, and the draft has gained significant merit. Therefore, I may approve the current version of the article.

Is the study design appropriate and does the work have academic merit?

Yes

Is the work clearly and accurately presented and does it cite the current literature?

Yes

If applicable, is the statistical analysis and its interpretation appropriate?

Partly

Are all the source data underlying the results available to ensure full reproducibility?

Yes

Are the conclusions drawn adequately supported by the results?

Yes

Are sufficient details of methods and analysis provided to allow replication by others?

Yes

Reviewer Expertise:

Biomaterials engineering, especially development and characterization of bone cements: polymeric (PMMA, pHEMA), ceramic (MPC) and biocomposite-based.

I confirm that I have read this submission and believe that I have an appropriate level of expertise to confirm that it is of an acceptable scientific standard.

# Reviewer report: Reviewer response for version 1

**DOI:** 10.21956/openreseurope.18016.r37218

**Published:** 2024-02-20

**Authors:** Jakub Szabelski

**Affiliations:** 1 Lublin University of Technology, Lublin, Poland

Overall, the paper is quite interesting as it explores the results of research on the mechanical properties of PMMA bone cement with the addition of linoleic acid to lower the elastic modulus, making it more suitable for patients with osteoporosis. However, considering the wide range of topics covered by the journal where the paper was submitted, I suggest providing a more thorough explanation of the background behind the research. A broader literature review is necessary, especially since the initial chapters of the introduction pose several unanswered questions. For instance:

1. What other compounds or naturally occurring contaminants were previously examined, impacting the Young modulus and general mechanical properties of PMMA bone cements? How do specific additives influence the cement and alter its properties? Consider references such as cellulose (Ref 6), polydioxanone (Ref 7), polyethylene (Ref 8), TCP, HA, etc., as well as contaminants like saline solution and blood (Ref 9, Ref 10).
2. Why choose linoleic acid as the next additive? What is linoleic acid, and what are its properties? Refer to sources such as (Ref 1, Ref 2, Ref 3).

Some questions can be answered in one simple sentence, by referencing papers, including those authored by the paper's researchers, while others may require additional explanations within the text.

 With regard to the statistical analysis of the Young's modulus results, it may be worthwhile to mention in the paper that due to observed, quite obvious differences between obtained values, a detailed statistical analysis may not be necessary. Alternatively, a simple ANOVA comparison could be conducted to definitively establish the significance of the changes.

 In the discussion, refrain from directly comparing results that have standard deviation presented as ±. Such results cannot generally be compared, unless it's clarified that the reference is to mean values.

 Consider presenting all three curves from Figure 1 on one chart, and perhaps create an additional chart displaying only the three Weibull curves (without 95% bounds and raw data) for clearer visual comparison. Or maybe include those 95% bounds but use different line style?

 The discussion mainly focuses on comparing the authors' results, which is valid. However, it would be beneficial to go further and explain why these specific results may have been obtained. In the introduction authors mentioned of porosity and plasticization of cement. At this point, it would have been helpful to address these issues based on the relevant literature (e.g., Ref 4, Ref 5). Additionally, please present results from similar research and their observations.

 Consider removing the "Commercially Available cement" entry in Table 2, as it lacks specificity, and even the author of reference #34 does not specify which cement is being referred to.

 One notable strength of the paper is the decision to release a complete set of raw data, videos, and Excel spreadsheets, adding significant value to the research.

Is the study design appropriate and does the work have academic merit?

Yes

Is the work clearly and accurately presented and does it cite the current literature?

Partly

If applicable, is the statistical analysis and its interpretation appropriate?

Partly

Are all the source data underlying the results available to ensure full reproducibility?

Yes

Are the conclusions drawn adequately supported by the results?

Yes

Are sufficient details of methods and analysis provided to allow replication by others?

Yes

Reviewer Expertise:

mechanical properties of bone cements, biomaterials,

I confirm that I have read this submission and believe that I have an appropriate level of expertise to confirm that it is of an acceptable scientific standard, however I have significant reservations, as outlined above.

## References

[1] : Advances in research on microbial conjugated linoleic acid bioconversion. *Prog Lipid Res* .2023;93: 10.1016/j.plipres.2023.101257 101257 10.1016/j.plipres.2023.101257 37898352

[2] : The effect of conjugated linoleic acids on inflammation, oxidative stress, body composition and physical performance: a comprehensive review of putative molecular mechanisms dust. *https://doi.org/10.1186/s12986-023-00758-9* .2023;10.1186/s12986-023-00758-9PMC1046684537644566

[3] : The Effect of Conjugated Linoleic Acid Supplementation on Densitometric Parameters in Overweight and Obese Women—A Randomised Controlled Trial dust. *https://doi.org/10.3390/medicina59091690* .2023;10.3390/medicina59091690PMC1053768037763810

[4] : Statistical distribution of micro and macro pores in acrylic bone cement- effect of amount of antibiotic content dust. *https://doi.org/10.1016/j.jmbbm.2023.106297* .2023;10.1016/j.jmbbm.2023.10629738100980

[5] : Estimation of Porosity Effect on Mechanical Properties in Calcium Phosphate Cement Reinforced by Strontium Nitrate Nanoparticles: Fabrication and FEM Analysis dust. *https://doi.org/10.1007/s13369-023-08050-x* .2023;

[6] : Pressure-engineered electrophoretic deposition for gentamicin loading within osteoblast-specific cellulose nanofiber scaffolds dust. *https://doi.org/10.1016/j.matchemphys.2021.125018* .2021;

[7] : Nanosilver-loaded PMMA bone cement doped with different bioactive glasses – evaluation of cytocompatibility, antibacterial activity, and mechanical properties dust. *https://doi.org/10.1039/D1BM00079A* .2021;10.1039/d1bm00079a33704333

[8] : The effect of ultra-high molecular weight polyethylene fiber on the mechanical properties of acrylic bone cement dust. *https://doi.org/10.1007/s10965-006-0006-2* .1997;

[9] : Evaluation of the Effect of Selected Physiological Fluid Contaminants on the Mechanical Properties of Selected Medium-Viscosity PMMA Bone Cements dust. *https://doi.org/10.3390/ma15062197* .2022;10.3390/ma15062197PMC895135735329650

[10] : Effect of Physiological Fluids Contamination on Selected Mechanical Properties of Acrylate Bone Cement dust. *https://doi.org/10.3390/ma12233963* .2019;10.3390/ma12233963PMC692697931795371

# Response

**Published:** 2024-04-16

**Authors:** Salim Ghandour

**Affiliations:** Department of Material Science and Engineering, Uppsala University, Uppsala, Uppsala County, Sweden

The authors would like to thank the reviewer for taking the time to review our study. Please find below a point by point response and changes in-line with the reviewers comments:

- The authors thank the reviewer for this comment. We have added information on different types of additives that have been used previously for the purpose of reducing the mechanical properties of PMMA cements. A more in-depth report on the effect of each additive would certainly be very interesting, but we cannot find a suitable review paper on this in the literature.
- The authors thank the reviewer for this comment. We have included in the introduction several advantages that linoleic acid has, such as a reduction of polymerization temperature, with appropriate references. The motivation of the choice has also been backed with the advancement of this type of cement in the value chain.
- The authors thank the reviewer for this comment. We have included a statistics section in the materials and method section that outlines that we have perform a t-test to observe any statistically significant differences for the quasi-static tensile properties.
- The authors thank the reviewer for this suggestion. We have specified that we are referring to the mean values.
- The authors thank the reviewer for this suggestion. We have attempted to create such graphs however we believe that these present no added value as some of the curves would not be adequately visible due to the vast differences in the ranges of the different fatigue cycles.
-   The authors thank the reviewer for this comment. We have included an explanation of the mechanism of action for linoleic acid in the discussion section for clarity. To the authors’ knowledge only 1 study by Weightman et al. has studied the tensile properties of a low modulus cement, although for hip replacement fixation – that has been included in the discussion section. All other literature at present contains mechanical testing in compression as it is a requirement for the ASTM F451.
- The authors thank the reviewer for this suggestion. Although we agree the naming is vague, due to the limited literature on this topic we have decided to keep this reference as it adds value to the results found in this paper. We have however added to the table that it is ‘unspecified’.
- Thank you for this comment, we agree that this increases the value of research results in general.

# Reviewer report: Reviewer response for version 1

**DOI:** 10.21956/openreseurope.18016.r37217

**Published:** 2024-01-24

**Authors:** Marcin Wekwejt

**Affiliations:** 1 Gdansk University of Technology, Gdansk, Poland

This manuscript, Quasi-static and dynamic mechanical properties of a low-modulus bone cement for spinal applications, is an interesting paper focusing on PMMA bone cement modification with linoleic acid and its potential for tailoring cement stiffness, especially for percutaneous discoplasty application. The paper is well-thought, contains valuable results in the field of quasi-static tension and tension-compression fatigue properties of composite cement and is completely original. However, there are still several points that should be improved:

-  The title is not precise.
-  In the Introduction, the main results from papers 27-29 should be generally presented to the reader. In particular, it should be explained why such a modification and its concentration of 12% were chosen?
-  The terms *in vivo* and *in vitro* should be written in italics.
-  In Materials and Methods, please add the number of repetitions of a given group. A description of the statistical method should also be included.
-  The results for the Young's Module and UTS should also be presented graphically - this makes it easier for the reader to check the most important content.
-  The Results chapter is relatively short - it should contain a description of the obtained results (currently described in the Discussion).
-  A paragraph about the limitation would be useful in the Discussion.
-  It is also necessary to explain in more detail how this modification affects the mechanical properties of cement, especially from a structural perspective.

Is the study design appropriate and does the work have academic merit?

Yes

Is the work clearly and accurately presented and does it cite the current literature?

Yes

If applicable, is the statistical analysis and its interpretation appropriate?

Partly

Are all the source data underlying the results available to ensure full reproducibility?

Yes

Are the conclusions drawn adequately supported by the results?

Yes

Are sufficient details of methods and analysis provided to allow replication by others?

Yes

Reviewer Expertise:

Biomaterials engineering, especially development and characterization of bone cements: polymeric (PMMA, pHEMA), ceramic (MPC) and biocomposite-based.

I confirm that I have read this submission and believe that I have an appropriate level of expertise to confirm that it is of an acceptable scientific standard, however I have significant reservations, as outlined above.

# Response

**Published:** 2024-04-16

**Authors:** Salim Ghandour

**Affiliations:** Department of Material Science and Engineering, Uppsala University, Uppsala, Uppsala County, Sweden

The authors thank the reviewer for the time spent on reviewing our paper and the valuable comments. In line with the reviewer’s suggestion, the authors have modified the manuscript as such:

- The title to ‘Quasi-static and dynamic mechanical properties of a linoleic acid-modified, low-modulus bone cement for spinal applications’.
- The authors thank the reviewer for this question. We have specified the results of these references in the introduction. Further, we have clarified that the volume percentage of linoleic acid is based on these previous studies, chosen to match the properties of vertebral cancellous bone.
- In vivo and in vitro are now formatted in italics.
- The authors thank the reviewer for this suggestion. We have included the number of samples used per test. Further, we have included a statical analysis section elaborating on the statistics used in this paper to analyse the quasi-static test data. The fatigue data was analysed as per the standard, and already described under the fatigue testing section in the materials and methods section.
- The authors thank the reviewer for this suggestion. We have included a figure of these results, including the statistical analysis results.
- The authors thank the reviewer for this comment. All the obtained results that were described in the Material and Methods section have been portrayed in the results section, we have also added the suggested figure. Some additional descriptions have been added. The discussion section explains the results and set them in perspective to other work in the literature, as is customary. If the reviewer has any specific suggestions on how the results section can be further expanded on we are happy to receive these comments.
- The authors thank the reviewer for this comment. We agree and have added a paragraph on limitations and future work to the discussion section.
- The authors thank the reviewer for this comment. The mechanism behind the reduction of mechanical properties has been added to both the introduction section and the discussion section.
